# Malaria in Children Adopted from the Democratic Republic of the Congo

**DOI:** 10.3201/eid2304.161777

**Published:** 2017-04

**Authors:** Elena Chiappini, Sara Sollai, Maurizio de Martino, Luisa Galli

**Affiliations:** Meyer University Hospital, Florence University, Florence, Italy

**Keywords:** malaria, internationally adopted children, screening, zoonoses, parasites, Democratic Republic of the Congo, Italy

## Abstract

Data are lacking regarding asymptomatic and symptomatic malaria prevalence in internationally adopted children. Among 20 children from Democratic Republic of the Congo evaluated in Florence, Italy, in April 2016, malaria prevalence was 80%; 50% of infected children had symptomatic malaria. Adopted children from areas of high malaria endemicity should be screened for malaria.

The Democratic Republic of the Congo (DRC) banned adoption of children by parents from other countries in 2013. In February 2016, the ban was removed, and several hundred children were allowed to join families in Europe and in the United States. The first group of children from DRC arrived in Italy in April 2016, and 20 children were referred to the Center for Internationally Adopted Children at Meyer University Hospital, Florence, Italy. All children underwent the standard infectious disease screening tests recommended by the American Academy of Pediatrics, including those for tuberculosis (by tuberculin skin test and interferon-γ release assay) and intestinal parasites (by fecal testing for ova, parasites, and antigen test for *Giardia* spp.) and serologic tests for *Toxocara canis, Strongyloides* spp., hepatitis B and C viruses, HIV-1/2 viruses, and *Treponema pallidum* (syphilis) (*1*).

Eight children who were exhibiting fever were admitted to the hospital and received a diagnosis of malaria soon after their arrival in Italy. For malaria testing, PCR and microscopy were performed on thin and thick smears. Parasitemia level was determined by counting the parasitized erythrocytes among the 500–2,000 erythrocytes on the thin smear and calculating the percentage.

The remaining children from DRC were screened, and another 8 children were found to be infected. Thus, malaria was diagnosed in 16 children (10 were boys; median age 7 years [range 4–10 years]), and malaria prevalence was 80% (16/20). *Plasmodium falciparum* infection was documented in 15 cases, whereas a mixed infection (*P.* *falciparum* and *P. ovale*) was observed in 1 child. All children underwent treatment with intravenous quinine plus artesunate or oral dihydroartemisinin/piperaquine (*2*). Intravenous treatment was administered to 1 child who had severe malaria (generalized seizures) and to 5 children with a parasitemia level >2% or who exhibited vomiting and therefore were unable to take oral medications reliably (*2*). Because intravenous artesunate is unlicensed in Europe but is available in our center, we obtained written informed consent for its use from the patients’ parents before administration. The study received approval by Meyer University Hospital Ethics Committee. 

Other studies have assessed the prevalence of malaria in internationally adopted children. Among a population of 182 children in France, when Blanchi et al. screened for the children originating from malaria-endemic zones who were exhibiting fever, splenomegaly, or both for malaria, they found 2 infected children (*3*). More recently, Adebo et al. screened 52 children arriving in the United States from Ethiopia for malaria and reported 7 (13.5%) children with asymptomatic malaria (*4*). These authors suggested that screening be conducted of children coming from malaria-endemic areas with noted risk factors, such as splenomegaly (*4*). Anemia (hemoglobin levels <11 g/dL) could be another risk factor for malaria but, because it is common in this population, it was not considered in the study by Adebo et al. (*4*). However, in our population, we found that tests for parasitemia were positive for 6 children without splenomegaly or hepatomegaly and for 1 child who had neither anemia (<11 g/dL), hepatomegaly, nor splenomegaly. Therefore, using clinical features to select children who should undergo the screening may be challenging.

Given the paucity of literature data regarding malaria prevalence in internationally adopted children, testing by PCR, microscopy, or both, followed by treatment of infected children, would be preferable to the empiric treatment, considering the costs and possible adverse effects of antimarial drugs. Moreover, the preferable screening strategy is not apparent. We did not observe any discrepancy between microscopy and PCR results; however, a higher sensitivity by PCR has been reported (*4*,*5*). In contrast, some experts prefer testing by microscopy examination because PCR techniques are not sufficiently standardized or validated to be used for routine clinical diagnosis (*2*).

In our dataset, malaria prevalence was substantially higher than that previously reported (*4*). This finding may be due to the particular situation of these children and to orphanage conditions (i.e., lack of mosquito nets). Moreover, it should be noted that, to date, 3 countries— DRC, Nigeria, and India—account for 40% of all estimated malaria cases in the world (*6*). Also, a high prevalence of asymptomatic malaria in DRC has been reported, in ≈15% of children (*7*,*8*).

Our results should be interpreted with caution, given the small dataset, but they should alert pediatricians regarding the importance of assessing malaria risk in children who have been adopted internationally. The degree of malaria endemicity in the child’s area of origin may be considered in the decision to screen asymptomatic children adopted in non–malaria-endemic countries. In particular, children who come from areas of high malaria endemicity, such as DRC, deserve a careful screening, even in the absence of any sign or symptom.
